# MambaOSR: Leveraging Spatial-Frequency Mamba for Distortion-Guided Omnidirectional Image Super-Resolution

**DOI:** 10.3390/e27040446

**Published:** 2025-04-20

**Authors:** Weilei Wen, Qianqian Zhao, Xiuli Shao

**Affiliations:** VCIP, College of Computer Science, Nankai University, Tianjin 300350, China; zhaoqian9708@163.com

**Keywords:** omnidirectional image super-resolution, Mamba, spatial frequency, mutual information

## Abstract

Omnidirectional image super-resolution (ODISR) is critical for VR/AR applications, as high-quality 360° visual content significantly enhances immersive experiences. However, existing ODISR methods suffer from limited receptive fields and high computational complexity, which restricts their ability to model long-range dependencies and extract global structural features. Consequently, these limitations hinder the effective reconstruction of high-frequency details. To address these issues, we propose a novel Mamba-based ODISR network, termed MambaOSR, which consists of three key modules working collaboratively for accurate reconstruction. Specifically, we first introduce a spatial-frequency visual state space model (SF-VSSM) to capture global contextual information via dual-domain representation learning, thereby enhancing the preservation of high-frequency details. Subsequently, we design a distortion-guided module (DGM) that leverages distortion map priors to adaptively model geometric distortions, effectively suppressing artifacts resulting from equirectangular projections. Finally, we develop a multi-scale feature fusion module (MFFM) that integrates complementary features across multiple scales, further improving reconstruction quality. Extensive experiments conducted on the SUN360 dataset demonstrate that our proposed MambaOSR achieves a 0.16 dB improvement in WS-PSNR and increases the mutual information by 1.99% compared with state-of-the-art methods, significantly enhancing both visual quality and the information richness of omnidirectional images.

## 1. Introduction

In recent years, omnidirectional imaging technology has garnered substantial attention from both academic and industrial sectors, driven by the rapid development of immersive applications such as virtual reality (VR) and augmented reality (AR). These applications demand high-quality visual content, which typically requires resolutions of 4K or even 8K to ensure optimal viewing experiences. However, capturing such high-resolution omnidirectional images usually involves sophisticated and costly imaging equipment. Due to the resolution limitations of commonly available devices, ODISR has emerged as an essential research direction in this field. From an information theory perspective, low-resolution (LR) omnidirectional images exhibit diminished information content, resulting from sampling limitations, quantization errors, and sensor noise inherent in the acquisition process. Super-resolution (SR) techniques employ deep learning models to approximate high-frequency details through learned statistical priors, aiming to enhance visual fidelity.

An omnidirectional image exhibits fundamental differences from a traditional 2D image in geometric representation and storage formats. The most common formats, i.e., equirectangular projection (ERP) and cubemap projection (CMP), are designed to represent spherical content on planar surfaces. ERP maps the entire spherical view onto a rectangular grid, often introducing latitudinal stretching artifacts near polar regions, while CMP divides the sphere into six cube faces, reducing distortions but introducing face-boundary discontinuities. These unique properties pose challenges for effective feature extraction.

To address these distinctive characteristics inherent in omnidirectional images, researchers have developed dedicated SR architectures that explicitly model spherical geometry and distortion patterns. LAU-net [1] employs a latitude-based tiling strategy and implements independent processing pipelines to address varying distortion levels. However, such strategies are at risk of introducing boundary artifacts across adjacent tiles. In contrast, OSRT [2] incorporates a transformer-based architecture with a distortion-aware module that performs geometric rectification. Although OSRT demonstrates superior performance over previous ODISR approaches, its transformer-based design incurs quadratic complexity scaling with token size, presenting significant computational bottlenecks when processing high-resolution omnidirectional content.

**Figure 1:**
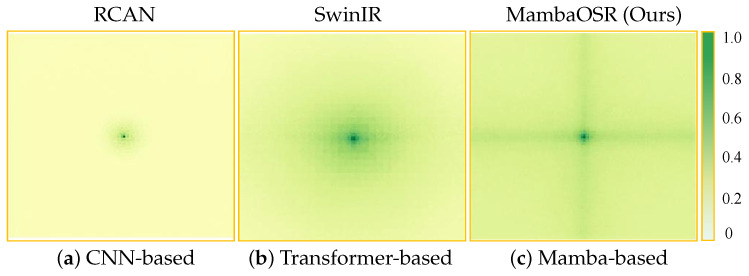
Comparison of Effective Receptive Field (ERF) for (**a**) the CNN-based method, RCAN [3], (**b**) the transformer-based model, SwinIR [4], and (**c**) the proposed Mamba-based network, MambaOSR. A larger ERF is represented by a wider distribution of dark areas. The proposed MambaOSR achieves the largest ERF, demonstrating its superior global context modeling capability.

Existing ODISR methods mainly leverage either CNN-based or transformer-based architectures to formulate their solutions. As shown in Figure 1, the CNN-based model, RCAN [3], is constrained by their local receptive fields, limiting their ability to model self-similarity patterns. This requirement is crucial in SR tasks, where pixel reconstruction relies on contextual information. In ODISR, this dependency intensifies due to geometric distortions demanding strong spatial awareness. Transformers provide global context but face computational challenges due to their quadratic complexity with token length. This motivates a fundamental question: How to design an efficient architecture that jointly addresses long-range dependency modeling in ODISR while enhancing information entropy through effective feature aggregation. Recent advances in state space models (SSMs), with their linear complexity scaling and global context modeling capabilities, present a promising direction to tackle this challenge.

The Mamba architecture [5] integrates SSMs with MLPs through an input-dependent selective scanning mechanism, enabling context-aware feature selection during long-range modeling while preserving task-specific representations. This design enhances the model’s capacity for discriminative representation learning, making it well suited for addressing key ODISR challenges such as the correction of geometric distortions and the preservation of high-frequency details. Therefore, we have adopted Mamba as the backbone architecture. Building upon this foundation, MambaIR [6] and MambaIRV2 [7] have established new benchmarks in image restoration by leveraging Mamba’s efficient long-range modeling capabilities. Despite the success of Mamba in sequence modeling tasks, adapting this architecture directly to high-resolution omnidirectional images remains challenging due to inherent spherical distortions in ERP representations. Addressing these distortions requires explicit modeling and expanded receptive fields to effectively capture the complex relationships within spherical content.

To bridge this gap, we introduce MambaOSR, the first Mamba-based framework for ODISR that integrates spatial-frequency modeling with distortion-aware scanning through a dual-path architecture. Our framework mitigates spherical distortions and utilizes spatial-frequency mixture representations to improve omnidirectional image reconstruction. Specifically, the key component of MambaOSR is the spatial-frequency Mamba block (SFMB), which contains a spatial-frequency visual state space model (SF-VSSM) and a distortion-guided module (DGM). The SF-VSSM integrates a frequency-aware module (FAM) with Mamba to adaptively extract frequency-domain information critical for ODISR. By effectively combining spatial- and frequency-domain features, the module enhances the long-range modeling capability of the Visual State Space Model (VSSM), resulting in more accurate feature reconstruction. Furthermore, to tackle distortions inherent to omnidirectional images, particularly along the latitudinal direction, we introduce the DGM. This module employs learned affine transformation parameters to dynamically adjust image feature mappings, thereby mitigating geometric distortions and improving reconstruction quality. Finally, the proposed multi-scale feature fusion module (MFFM) efficiently aggregates multi-resolution features to preserve fine-grained details in reconstructed images. Our method mitigates spherical distortion artifacts and enhances global context modeling, achieving high-fidelity reconstruction with linear computational complexity.

The main contributions are summarized as follows:

(1) We introduce state space models into ODISR and propose an efficient network named MambaOSR. By leveraging the strong global modeling capabilities of the Mamba architecture, our method effectively captures long-range dependencies, significantly improving reconstruction quality. Extensive experiments validate the superior performance of MambaOSR compared to existing methods.
(2) To further enhance global context modeling, we propose an SF-VSSM. Specifically, the SF-VSSM integrates an FAM with the VSSM to adaptively exploit frequency-domain information beneficial to the ODISR task. This integration enhances the model’s ability to capture global structural features and improves the reconstruction accuracy.
(3) To address the degradation of image quality caused by geometric distortions inherent in omnidirectional imaging, we introduce a DGM. The DGM leverages distortion map priors to adaptively fuse the information of geometric deformation, effectively suppressing distortion artifacts. Additionally, we design an MFFM to integrate features across multiple scales, further strengthening the model’s representation capability and enhancing reconstruction performance.

## 2. Related Works

In this section, we revisit classical 2D SR and panoramic super-resolution methods, alongside SSM and Fourier transform techniques relevant to this work. Specifically, in the subsection on single-image super-resolution (SISR), we categorize the approaches into four groups: convolutional neural network (CNN)-based SISR methods, generative adversarial network (GAN)-based SISR methods, transformer-based SISR methods, and diffusion-based SISR methods.

### 2.1. Single-Image Super-Resolution

SISR is a crucial image reconstruction task aimed at reconstructing high-resolution (HR) images from low-resolution (LR) inputs. With the evolution of deep learning techniques, SISR techniques have gone through various approaches, including convolutional neural network CNN-based, GAN-based, transformer-based, and, more recently, diffusion-based models. Each approach offers unique characteristics and has demonstrated significant improvements across different application scenarios.

#### 2.1.1. CNN-Based SISR Methods

CNN-based SISR methods [3,8,9,10] are among the earliest proposed approaches. A notable method is SRCNN [8], which utilizes standard convolutional layers to extract features and upscale LR images. It marks one of the pioneering deep learning models for SISR. Subsequently, RCAN [3] improved the model’s ability to capture fine details by integrating a residual channel attention mechanism. Building on this, HAN [9] introduced a holistic attention mechanism to enhance detail restoration. Although these methods demonstrate commendable performance in reconstructing complex structures and exhibit high computational efficiency, they often struggle to generate fine texture details.

#### 2.1.2. GAN-Based SISR Methods

The application of generative adversarial networks [11] in SISR primarily focuses on generating realistic image textures. SRGAN [12] was the first model to apply GANs for super-resolution, using adversarial training to generate high-frequency details and enhance the visual quality of reconstructed images. Following this, ESRGAN [13] introduced dense skip connections and feature pooling, resulting in sharper and more detailed HR images. GAN-based SISR methods show clear advantages in visual quality, particularly in generating complex textures. However, they may also introduce artifacts or unrealistic details.

#### 2.1.3. Transformer-Based SISR Methods

Recently, transformer architectures have demonstrated strong modeling capabilities in SISR tasks. IPT [14] is one of the first SISR models to incorporate the transformer architecture. It enhances the model’s generalization ability through multi-task learning and large-scale pre-training. SwinIR [4] utilizes the Swin transformer, segmenting images into local windows to improve feature extraction accuracy while effectively reducing computational overhead. HAT [15] employs a hierarchical aggregation mechanism to fuse multi-scale information, significantly enhancing the quality of image detail reconstruction. These methods excel in capturing long-range dependencies and complex textures but often require significant computational resources.

#### 2.1.4. Diffusion-Based SISR Methods

Recently, diffusion models have gained attention in generative tasks, with SR3 [16] being a representative model that reconstructs HR images from LR inputs through a progressive denoising process. SRDiff [17] further optimizes the diffusion process, improving the speed and quality of image generation. Methods like ResShift [18] explore more efficient variants of diffusion models for image generation. Although these approaches have demonstrated significant potential in generating high-quality images, their iterative denoising processes can lead to longer inference times.

The evolution of SISR methods from CNNs to GANs, transformers, and diffusion models showcases continuous improvements in reconstruction quality, computational efficiency, and detail preservation. CNN-based methods are well suited for real-time applications, GANs excel in visual perception, and transformers and diffusion models offer significant advantages in high-quality image reconstruction. Future developments in SISR technology may further integrate the strengths of these diverse approaches to achieve higher quality and efficiency in SISR tasks.

### 2.2. Omnidirectional Image Super-Resolution (ODISR)

ODISR aims to enhance the resolution of omnidirectional images (ODIs), which are typically captured using omnidirectional cameras. Raw ODIs often appear in fisheye projections and are transformed into 2D images through different stitching techniques. Among these, ERP is the most commonly employed format for storing ODIs. Given its extensive applications in VR, AR, autonomous driving, and remote monitoring, ODISR has increasingly attracted research interest.

Early methods in this domain extended classical SISR techniques to tackle the unique challenges posed by ODIs. For instance, 360-SS [19] introduced an SISR model augmented with a spherical loss function, providing a straightforward approach to solving the ODISR problem. Similarly, Nishiyama et al. [20] incorporated distortion map information into the SISR framework to address deformation distortions in ODISR tasks. Although these methods offered initial solutions, they often fell short of fully leveraging the intrinsic characteristics of ODIs, limiting their effectiveness in addressing distortion-related artifacts.

Recently, researchers have increasingly focused on the challenge of uneven pixel density in ODIs. LAU-Net [1] addresses this issue by hierarchically partitioning ODIs based on latitude differences, enabling the network to process distinct latitude strips at varying levels. Building upon this framework, LAU-Net+ [21] introduces a multi-level pyramid network along with feature enhancement modules to further improve the performance of ODISR models. Despite their advancements, the hierarchical processing strategy, which relies on non-overlapping image patches, leads to noticeable artifacts at the boundaries of latitude strips in reconstructed ERP images. SphereSR [22] introduces a novel feature extraction module based on an icosahedral representation to effectively capture spherical features, utilizing a Spherical Local Implicit Image Function to predict RGB values in spherical coordinates. This approach enables the generation of continuous spherical images and supports flexible HR reconstruction across various projections. To explicitly address deformation distortions in ERP images, OSRT [2] incorporates a distortion-aware transformer with deformable convolutions. Similarly, OPDN [23] adopts a two-stage framework that integrates a position-aware deformable module alongside a frequency fusion module. BPOSR [24] leverages the complementary geometric properties of ERP and CMP projections through a dedicated attention mechanism, significantly improving the performance of ODISR. Cai et al. [25] proposed a spherical pseudo-cylindrical representation with adaptive latitude sampling and viewport-based loss for ODISR, which demonstrated promising performance while maintaining model-agnostic characteristics. Li et al. [26] presented OmniSSR, a zero-shot approach leveraging stable diffusion with tangent projections for ODISR, improving fidelity and realism without training. Yang et al. [27] adapted 2D planar SR models via low-rank adaptation (LoRA) for real-world ODISR, effectively reducing parameters and computational costs. Yang et al. [28] introduced GDGT-OSR with distortion-aware attention and dynamic feature aggregation guided by latitude-variant distortion modulation, achieving superior ODISR performance. Despite the progress made by existing ODISR methods, challenges remain, particularly in addressing ERP distortions and reducing computational complexity.

### 2.3. State Space Model (SSM)

In recent years, state space models grounded in control theory have achieved significant advances in deep learning. Compared to transformers, these models deliver competitive performance in long-range modeling tasks, with computational complexity scaling linearly with sequence length. Gu et al. [29] have established this linear growth advantage of SSMs when handling long sequence data. The introduction of the Mamba model, which outperforms transformers in natural language processing tasks, has garnered considerable attention. Due to its outstanding performance, the Mamba model has expanded rapidly into the computer vision domain, showing promising progress in tasks such as image restoration [6,7,30], image classification [31], and object detection [32]. However, the application of the Mamba model in foundational vision tasks remains in its early research stages, particularly in the context of ODISR, which remains to be explored.

This paper presents a novel framework for ODISR built upon the Mamba model. The proposed approach enables a more comprehensive global exploration of omnidirectional images by operating within the spatial-frequency dual domain. By integrating frequency domain information into the Mamba model, our method effectively enhances the extraction of high-frequency details, significantly improving the model’s ability to capture global features and structural information.

### 2.4. Fourier Transform

The Fourier transform has demonstrated an exceptional ability to capture global information, leading to its widespread adoption in many computer vision tasks [33,34,35,36,37] for frequency-domain representation. Frequency-domain characterization methods have driven significant breakthroughs in low-level visual tasks. The DeepRFT framework [38] leverages the global receptive field properties of Fourier transforms to capture both high- and low-frequency components for image deblurring. Building on the fast Fourier transform, the fast Fourier convolution (FFC) framework [39] establishes efficient paradigms for frequency-domain computation. Subsequently, Lama [40] developed an image inpainting model using fast Fourier convolution layers, achieving remarkable performance in reconstructing missing large-scale regions, complex geometric structures, and high-resolution images. Zhou et al. [41] present SFINet++, a spatial-frequency dual-domain network with invertible neural operators and deep Fourier transforms for multi-modal image fusion, outperforming SOTA in deep SR techniques. In the field of remote sensing image SR and ODISR, Xiao et al. [42] propose FMSR, the first Mamba-based framework for remote sensing image SR that integrates frequency-assisted modules and multi-level feature fusion, achieving linear-complexity long-range dependency modeling through learnable adaptors. Additionally, TSFNet [43] introduces a two-stage spatial-frequency joint learning framework, incorporating an amplitude-guided phase adaptive filter module and cross-stage feature fusion mechanism, enabling progressive refinement for large-factor remote sensing image SR. FATO [44] leverages Discrete Cosine Transforms (DCTs), frequency self-attention mechanisms, and frequency loss functions to enhance the preservation of high-frequency details in ODISR tasks.

A Fourier transform maps images from the spatial to the frequency domain, enabling models to capture global frequency characteristics and periodic patterns in images. This property is crucial for addressing geometric distortions commonly found in omnidirectional images. Although frequency-domain information is valuable, it alone cannot fully capture the local features of complex distortions. To overcome this limitation, we propose a frequency-aware module that leverages the Fourier transform for global frequency feature extraction while capturing local spatial details through convolution operations. By integrating spatial- and frequency-domain features, this module can expand the model’s receptive field and enhance the perception of high-frequency information. As illustrated in Figure 1, our method achieves a broader receptive field compared to both CNN-based and transformer-based approaches. Moreover, the computational complexity of our method scales linearly with the input size, whereas transformer-based methods exhibit quadratic growth.

## 3. Methodology

**Figure 2:**
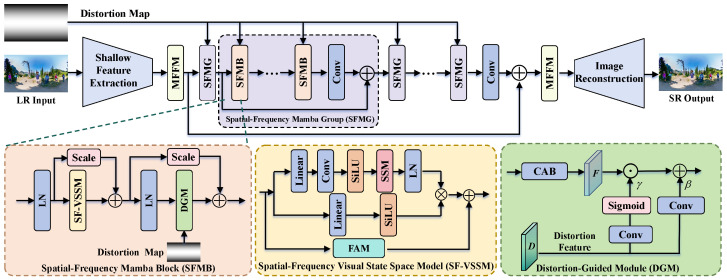
The overall architecture of the proposed MambaOSR. The key component of MambaOSR is the SFMB, consisting of the SF-VSSM and DGM. The SF-VSSM integrates Mamba with an FAM for adaptive frequency-domain feature extraction in ODISR, while the DGM enhances distortion representation to enable accurate texture recovery through distortion-aware learning. Additionally, the MFFM performs multi-scale feature integration to improve texture detail reconstruction through adaptive fusion mechanisms.

**Figure 3:**
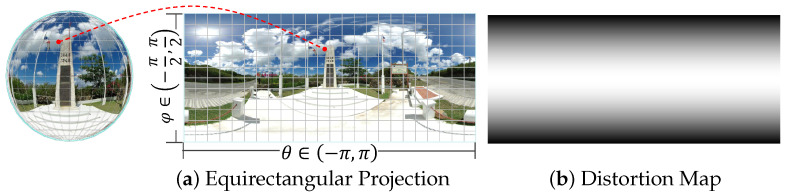
Illustration of the equirectangular projection (ERP) for 360° images. (**a**) Mapping of the spherical image to a 2D rectangular format with longitude (θ∈(−π,π)) and latitude (φ∈(−π/2,π/2)). (**b**) A distortion map indicating visual distortion exists in the ERP format, highlighting a non-uniform pixel distribution across the projection. The darker regions of the distortion map indicate a higher degree of distortion.

In this section, we first outline the overall framework of the proposed MambaOSR model, as shown in Figure 2. Subsequently, we introduce the commonly used ERP storage format for omnidirectional images, as shown in Figure 3, and we explain its fundamental principle. For ease of reference, the definitions of symbols and their meanings, which are discussed in Section 3, are listed in Table 1. We then elaborate on the key improvements introduced by Mamba for ODISR, which focus on three main components: the frequency-aware module (FAM) for capturing frequency-specific features, the distortion-guided module (DGM) for enhancing texture reconstruction in distorted regions, and the multi-scale feature fusion module (MFFM) for integrating features across multiple scales. Finally, we introduce the loss functions used in our approach.

### 3.1. Overview of MambaOSR

As depicted in Figure 2, we propose a Mamba-based network for ODISR that addresses the limited receptive fields hindering the reconstruction of details and the geometric distortions induced by the ERP. The proposed MambaOSR comprises three principal parts: the shallow feature extraction part, the deep feature extraction part, and the high-resolution image reconstruction part.

Given an LR image, shallow features are extracted via a 3 × 3 convolutional layer and enhanced through an MFFM for an adaptive feature representation. The processing procedure can be mathematically formulated as follows:

(1) $$F_{0}={\mathrm{MFFM}}_{1}\left(\mathrm{Conv}\left(I_{LR}\right)\right).$$

where ${\mathrm{MFFM}}_{1}$ represents the first instance of the MFFM, and Conv denotes the operation of the 3×3 convolutional layer.

Subsequently, $F_{0}$ undergoes deep feature extraction through multiple cascaded spatial-frequency Mamba groups (SFMGs), followed by a convolution layer for feature refinement.

(2) $$F_{n}=\mathrm{Conv}\left({\mathrm{SFMG}}_{n}\left({\mathrm{SFMG}}_{n-1}\left(\cdots {\mathrm{SFMG}}_{1}\left(F_{0}\right)\right)\right)\right),$$

where *n* denotes the number of SFMGs and $F_{n}$ is the output of the *n*-th SFMG. Each ${\mathrm{SFMG}}_{i}$ is composed of a cascade of spatial-frequency Mamba blocks (SFMBs):

(3) $$F_{i}=\mathrm{Conv}\left({\mathrm{SFMB}}_{m}\left({\mathrm{SFMB}}_{m-1}\left(\cdots {\mathrm{SFMB}}_{1}\left(F_{i-1}\right)\right)\right)\right)+F_{i-1},i=1,2,\ldots ,n$$

where $SFMB_{m}$ denotes the *m*-th SFGB in each SFMG. Finally, the refined features are further enhanced by another MFFM and upsampled through sub-pixel convolution to reconstruct the super-resolved output $I_{SR}$:

(4) $$I_{SR}=\mathrm{Conv}\left(\mathrm{Upsample}\left({\mathrm{MFFM}}_{2}\left(\mathrm{Conv}\left(F_{n}\right)+F_{0}\right)\right)\right).$$

### 3.2. Equirectangular Projection

The ERP implements a linear mapping scheme that transforms spherical coordinates into a 2:1 rectangular domain, discretized into an h×w pixel grid where *h* and *w* denote the height and width of the target resolution. Assuming that longitude and latitude are denoted by φ and θ, respectively, with $\left(\varphi ,\theta \right)\in \left[-\frac{\pi }{2},\frac{\pi }{2}\right]\times \left[-\pi ,\pi \right]$, the angular position(φ,θ) can be converted to Cartesian coordinates on a standard sphere $Q_{s}=\left(q_{s_{x}},q_{s_{y}},q_{s_{z}}\right)$ through the following relations:

(5)qsx=sin(φ)cos(θ),(6)qsy=sin(θ),(7)qsz=cos(φ)cos(θ).

As illustrated in Figure 3a, the ERP projection is widely used for omnidirectional image storage due to its computational efficiency, algorithmic simplicity, and minimal distortion in equatorial regions. However, the uniform planar sampling of ERP results in a non-uniform spherical sampling density, which increases near the poles. This variation leads to anisotropic stretching artifacts, with significant geometric distortions becoming more pronounced in polar regions.

The angular coordinates (θ,φ) on the unit sphere are transformed into planar coordinates (x,y) via the coordinate transformation functions of the ERP. This process is mathematically expressed as

(8) x=h(θ,φ)=θ,y=t(θ,φ)=φ.

h(·) and t(·) are coordinate transformation functions mapping spherical coordinates to the projection plane. As shown in Figure 3b, the original ODI exhibits geometric distortion when projected onto the ERP plane, characterized by latitudinal variation and hemispheric symmetry. This distortion motivates the formulation of a distortion map for low-resolution images. Given an LR image $I_{LR}\in R^{H\times W\times C_{in}}$ (where $H,W,C_{in}$ denote height, width, and channel dimensions, respectively), the corresponding distortion map $D\in R^{H\times W\times 1}$ is formulated as follows [45]:

(9) $$D\left(h,1:W\right)=\cos\left(\frac{h+0.5-\frac{H}{2}}{H}\pi \right),$$

where D(h,1:W) denotes the pixel stretching ratio from the ideal sphere to the 2D ERP image at height *h*. As shown in Figure 3, the distortion remains minimal in the equatorial region but intensifies toward higher latitudes. These distortions pose significant challenges for ODISR tasks.

### 3.3. Spatial-Frequency Visual State Space Model (SF-VSSM)

Previous ODISR methods often employ transformers to capture global dependencies via self-attention mechanisms for long-range response modeling. Despite their impressive performance, the high computational complexity of these methods hinders their efficiency in processing omnidirectional images. Inspired by the success of the VSSM in long-term modeling and aggregation with linear complexity, we pioneer its integration into ODISR tasks.

To enhance global information modeling, we introduce SF-VSSM, which contains two parallel modules: VSSM and FAM. The VSSM module, inherited from the Mamba architecture, performs dynamic state space modeling and visual feature extraction, while the proposed FAM enables effective spatial-frequency modeling, which significantly strengthens its ability to extract global information.

**Figure 4:**
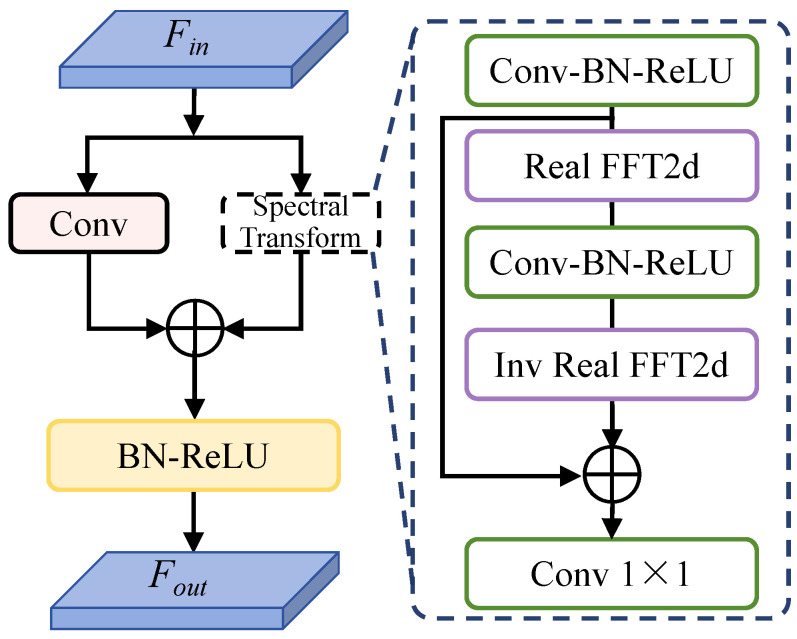
Overview of the FAM: The FAM processes features through parallel spatial and frequency branches. The frequency branch applies FFT-based spectral convolution and IFFT reconstruction, while the spatial branch uses conventional convolutions. Cross-domain feature fusion integrates spatial and frequency information to enhance high-frequency texture reconstruction.

As shown in Figure 4, the FAM adopts a dual-branch framework: a convolution branch for spatial feature extraction and a spectral transform branch for frequency-domain feature modeling. The spectral transform branch utilizes the Real Fast Fourier Transform (Real FFT) to represent image features. By exploiting the conjugate symmetry property of the Fourier transform, Real FFT processes only half of the spectrum (positive frequencies), significantly improving the computational efficiency. The spectral transform branch leverages its global receptive field to capture global context, while the convolution branch employs local kernels to model fine-grained details. By integrating their outputs, the dual-branch architecture expands the model’s receptive field and mitigates information loss that typically occurs during localized processing in reconstruction tasks. This fusion enhances the retention of critical image features and improves the structural similarity between the reconstructed image and the original high-resolution image. The specific operational architecture of the spectral transform is detailed in Figure 4. From the perspective of information theory, this integration increases the mutual information between the reconstructed image and its high-resolution counterpart, ensuring a more precise reconstruction.

(a) The *Real FFT2d* converts the feature tensor into the complex frequency domain
  $$\mathrm{RealFFT}2d:R^{H\times W\times C}\to C^{H\times \frac{W}{2}\times C},$$
  and concatenates its real and imaginary parts
  $$\mathrm{ComplexToReal}:C^{H\times \frac{W}{2}\times C}\to R^{H\times \frac{W}{2}\times 2C};$$
(b) Then, a convolutional block with activation and normalization processes the frequency spectrum information
  $$\mathrm{ReLU}\circ \mathrm{BN}\circ \mathrm{Conv}1\;\;\times \;\;1:R^{H\times \frac{W}{2}\times 2C}\to R^{H\times \frac{W}{2}\times 2C};$$
(c) The processed result is inversely transformed to restore spatial structure
  $$\mathrm{RealToComplex}:R^{H\times \frac{W}{2}\times 2C}\to C^{H\times \frac{W}{2}\times C},$$
  $$\mathrm{Inverse}\;\mathrm{Real}\;\mathrm{FFT}2d:C^{H\times \frac{W}{2}\times C}\to R^{H\times W\times C}.$$

Finally, the outputs of the convolution branch and spectral transform branch are fused through an addition operation.

In the SF-VSSM, the VSSM employs dynamic state space modeling to capture spatial-domain long-term dependencies, while FAM extracts spatial-frequency features through its dual-branch architecture for local detail and global context modeling. Here, LN represents the layer normalization operation. Additionally, we utilize a dynamic scaling factor to adaptively aggregate features based on their contextual significance. The output features *y* of the SF-VSSM can be formulated as follows:

(10) $$y=\alpha_{l}\cdot x_{l-1}+\mathrm{VSSM}\left(\mathrm{LN}\left(x_{l-1}\right)\right)+\mathrm{FAM}\left(\mathrm{LN}\left(x_{l-1}\right)\right).$$

### 3.4. Distortion-Guided Module (DGM)

Given the unique deformation characteristics inherent in ERP omnidirectional images, we propose a DGM to address these imaging-specific distortions. As shown in Figure 2, the DGM processes features through three main steps: First, the input feature *y* undergoes layer normalization to stabilize the feature distribution. Second, a channel attention block (CAB) captures global contextual information. Finally, the Spatial Feature Transform (SFT) layer is used to adaptively fuse the distortion map information with the processed features, enabling precise enhancement of locally significant deformation patterns. The overall process can be formulated as

(11) $$x_{l}=\alpha_{l+1}\cdot y+\mathrm{DGM}\left(\mathrm{LN}\left(y\right),condition\right),$$

where $\alpha_{l+1}$ is a learnable scaling factor that adaptively integrates feature representations in a data-driven manner. The detailed transformation within the DGM can be further expressed as

(12) DGM(LN(y),condition)=CAB(LN(y))·γ+β,

where the CAB processes normalized features, and the SFT layer modulates these features by applying condition-guided scaling factors γ=Sigmoid(Conv(condition)) and additive biases β=Conv(condition), as shown in Figure 2.

### 3.5. Multi-Scale Feature Fusion Module (MFFM)

To address the non-uniform spatial resolution distribution in ODIs, we propose an MFFM. By employing multi-scale feature representations, the proposed MFFM effectively mitigates information loss during the SR process while naturally adapting to the inherent spatial variations of ODIs.

**Figure 5:**
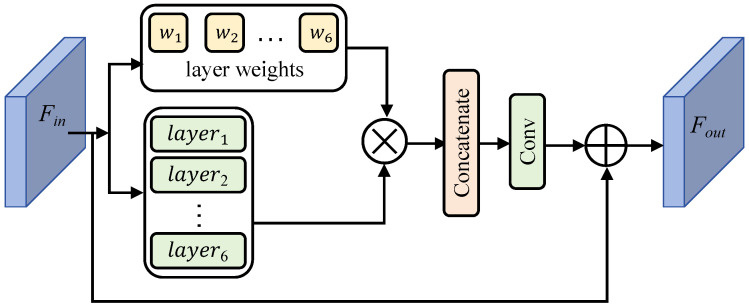
Illustration of the architecture of our proposed MFFM. The MFFM comprises six different convolutional layers with an adaptive weighting mechanism that dynamically fuses multi-scale features in an input-dependent manner.

As shown in Figure 5, the MFFM employs a parallel multi-branch architecture to capture multi-scale feature representations. The module consists of multiple sparse CNN layers organized in parallel: a 3 × 3 average pooling layer for global context modeling, separable convolutional layers with varying kernel sizes (3 × 3, 5 × 5, 7 × 7) for local feature extraction at different scales, and dilated convolutional layers for expanding the receptive field while maintaining resolution.

To dynamically route features across branches, we integrate a self-attention mechanism as the core of the routing process. This mechanism adaptively weights the contribution of each branch based on input characteristics, enabling context-aware spatial filtering.

Specifically, given the input features, the module first performs average pooling along spatial dimensions (*h* and *w*) to compute channel-wise statistics in *C* dimensions $z_{c}\in R^{C}$,

(13) $$z_{c}=\frac{1}{h\times w}\sum_{i=1}^{h}\sum_{j=1}^{w}x_{in}\left(i,j\right),$$

where $x_{in}\left(i,j\right)$ is the (i,j) position of the feature $x_{in}$. Then, according to two learnable weight matrices, $W_{1}\in R^{O\times T}$ and $W_{2}\in R^{T\times C}$, we generate weights $w_{out}$ for each branch:

(14) $$w_{out}=W_{2}\sigma \left(W_{1}z_{c}\right).$$

In addition, we perform zero-padding on the features to keep the size of the feature maps unchanged. The final output of the multi-scale fusion module can be formally expressed as

(15) $$x_{out}=f_{1\times 1}^{c}\left[\sum_{i=1}^{O}f_{b}\left(x_{out},w_{out}\right)\right]+x_{in},$$

where $f_{b}$ and *O* denote the operations of each branch and the total number of branches, respectively. $f_{1\times 1}^{c}$ represents a 1×1 convolution, and σ(·) is the ReLU activation function. [·] is the concatenation along the channel dimension. Using the MFFM, multi-scale information fusion can capture rich features in the omnidirectional images, providing more critical information for ODISR tasks.

### 3.6. Loss Function

The training process is guided by the $L_{1}$ loss between the super-resolved images $I_{SR}$ and their corresponding ground-truth counterparts $I_{GT}$, formulated as

(16) $$L_{sr}={∥I_{SR}-I_{GT}∥}_{1}$$

where ${∥\cdot ∥}_{1}$ denotes the $L_{1}$ norm.

## 4. Experiments

### 4.1. Experimental Configuration

In this section, we conduct SR experiments on omnidirectional images using multiple public datasets, including the ODI-SR dataset [1] and the SUN360 [46] omnidirectional dataset. The ODI-SR dataset contains 1200 training images and 100 testing ODIs; the SUN360 dataset contains 100 testing images. Our MambaOSR comprises six SFMGs for deep feature extraction, with each SFMG containing six SFMBs. Empirically, we set the dimension of the internal channel to c=96. The proposed model is implemented in PyTorch 2.0.1 with end-to-end training conducted on four NVIDIA A40 GPUs.

According to [2], we clean the training image pairs using the method proposed in OSTR. For the ×4, ×8, and ×16 experiments, we create HR/LR image pairs by directly downsampling the ERP images. We train the model using $L_{1}$ loss and optimize it with the Adam optimizer for efficient convergence, with an initial learning rate set to $1\times 10^{-4}$. In the ×4 experiments, the input image patch size is set to 64. For the ×8 and ×16 experiments, the LR image patch sizes are 32 and 16, respectively. The model is trained for 400,000 iterations. The learning rate is halved at 250,000 iterations to promote convergence. During the evaluation phase, we assess the model on the ODI-SR and SUN360 test sets, utilizing PSNR, SSIM [47], WS-PSNR [45], WS-SSIM [48], LPIPS [49] and Mutual Information (MI) [50] as evaluation metrics. Among these metrics, MI is employed to quantify the shared information between two images, providing a robust measure of their similarity.

To calculate MI, we first compute the entropy of individual images *X* and *Y*. The entropy values H(X) and H(Y) are defined as follows:

(17) $$H\left(X\right)=-\sum_{i}p\left(x_{i}\right)\log_{2}\left(p\left(x_{i}\right)+\epsilon \right),$$

(18) $$H\left(Y\right)=-\sum_{j}p\left(y_{j}\right)\log_{2}\left(p\left(y_{j}\right)+\epsilon \right),$$

where $p(x_{i})$ and $p(y_{j})$ are the probability distributions of the pixel intensity of images *X* and *Y*, respectively. To avoid numerical errors in logarithmic calculations, a small constant $\epsilon =10^{-9}$ is added. Next, joint entropy H(X,Y) is computed as

(19) $$H\left(X,Y\right)=-\sum_{i,j}p\left(x_{i},y_{j}\right)\log_{2}\left(p\left(x_{i},y_{j}\right)+\epsilon \right),$$

where $p(x_{i},y_{j})$ is the joint probability distribution, obtained by normalizing the joint histogram of images *X* and *Y*. Finally, Mutual Information MI(X,Y) is computed as

(20) MI(X,Y)=H(X)+H(Y)−H(X,Y).

This metric quantifies the shared information between images *X* and *Y*, making it useful for evaluating the similarity of reconstructed images in ODISR.

### 4.2. Evaluation Under ERP Downsampling

In the context of ERP downsampling, we compare nine representative SISR methods, including SRCNN [51], VDSR [52], LapSRN [53], MemNet [54], MSRN [55], EDSR [56], RCAN [3], HAT [15], MambaIR [6], and DRN [57], as well as five state-of-the-art (SOTA) ODISR algorithms: 360-SS [19], LAU-Net [1], SphereSR [22], OSRT [2], FATO [44] and BPOSR [24]. Our MambaOSR model is trained on the same ERP downsampling dataset to ensure fairness and consistency throughout the experimental process.

As shown in Table 2, our model demonstrates competitive performance for ×4, ×8, and ×16 upscaling factors on ERP downsampling datasets, ODI-SR and SUN360. Using the Mamba architecture as the backbone, our method significantly outperforms the baseline MambaIR across all test datasets. Compared to the state-of-the-art methods, our approach surpasses the Swin transformer-based BPOSR by 0.16 dB in PSNR and 0.0055 in SSIM on the SUN360 dataset for ×4 SR tasks. When evaluated against FATO, a novel frequency-based ODISR approach, our method shows notable improvements at ×4 and ×8 scaling factors, achieving a 0.23 dB gain on ODI-SR and a 0.16 dB gain on SUN360 for ×4 SR tasks, despite being slightly inferior at a ×16 scaling factor. Through quantitative analysis with the Mamba-based SISR method, MambaIR, and the state-of-the-art frequency-domain ODISR method, FATO, our method demonstrates superior overall performance in most scenarios, effectively validating its effectiveness and advancement.

In addition, we conducted experiments evaluating the LPIPS metric, which aligns closely with human perception and thus provides a more accurate measure of perceptual quality. As shown in Table 3, we compared our approach with several recent omnidirectional super-resolution algorithms. Our method demonstrates superior performance across multiple metrics, including PSNR, SSIM, and LPIPS. Specifically, on the LPIPS metric, our approach outperforms the latest state-of-the-art method, BPOSR, by margins ranging from 0.0095 to 0.0351 under various test conditions. This significant improvement highlights the ability of Mamba to extract global information and effectively integrate frequency-domain data, resulting in more perceptually accurate texture restoration.

Table 4 further highlights the superior MI performance of our model compared to other ODISR methods. On the SUN360 test set, our approach achieves MI improvements ranging from 1.99% to 37.95%. On the ODI-SR dataset, the MI gains range from 1.54% to 28.20%. As MI is a critical metric for quantifying structural similarity between super-resolved images and their high-resolution counterparts, these results highlight the robustness of our method. Complementary analyses using PSNR and SSIM confirm that our model achieves closer structural alignment with HR images than other approaches. These metrics provide a comprehensive evaluation of pixel-level accuracy and perceptual quality, highlighting the robustness of our approach.

**Figure 6:**
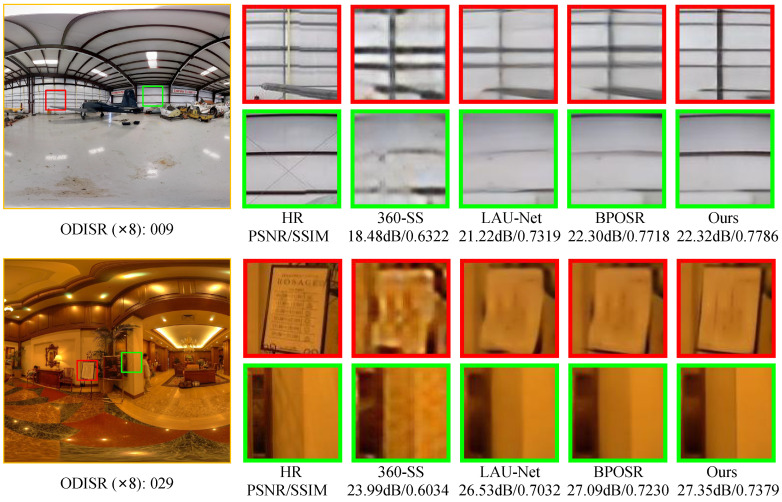
Visual comparisons of our MambaOSR with both SISR and ODISR methods on ODISR datasets on a ×8 scale. Our method demonstrates significant advantages over existing approaches in accurately reconstructing line structures. Zoom in for a better view.

**Figure 7:**
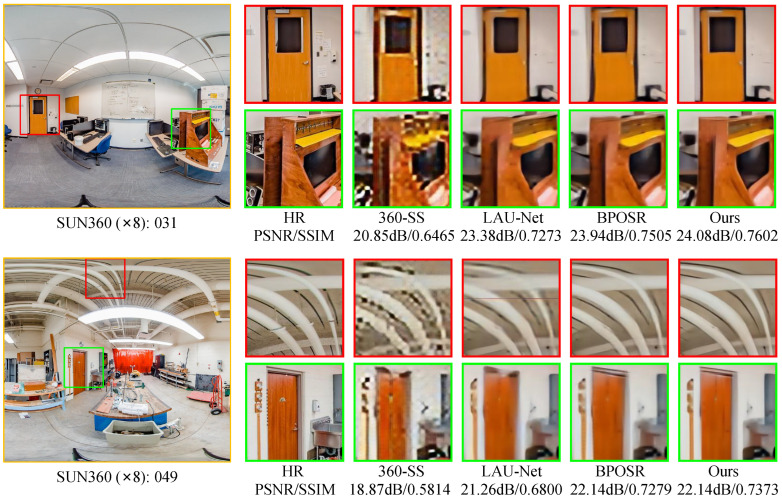
Visual comparisons of our MambaOSR with both SISR and ODISR methods on SUN360 datasets with a ×8 scaling factor. Our method shows significant improvements in reconstructing line structures compared to other approaches. Zoom in for a better view.

As demonstrated in Figure 6 and Figure 7, our approach consistently outperforms existing methods, including CNN-based and transformer-based ODISR approaches, in preserving fine texture details across both datasets. Our distortion-aware modeling strategy significantly enhances the uniformity and fidelity of reconstructed line segments by effectively incorporating geometric deformation priors. In comparison, methods such as BPOSR exhibit texture distortion due to their improper handling of such priors.

### 4.3. Model Efficiency

**Figure 8:**
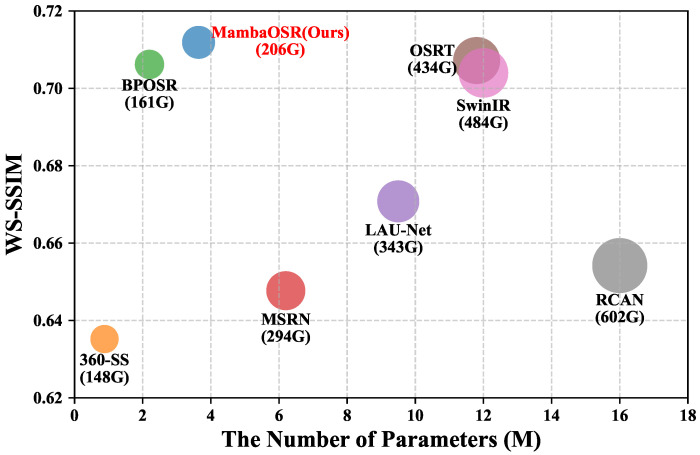
Comparison of WS-SSIM versus the number of parameters across various models on the SUN-SR test set with a ×8 upscaling factor. FLOPs (in G) are calculated based on LR input with a resolution of 256×128.

To comprehensively evaluate the performance of different methods, we compare the number of parameters and computational complexity of each method. The specific results are shown in Figure 8. Our method has a noticeable advantage over OSRT [2] and SwinIR [4] in the number of parameters and computation abilities, and it achieves significantly better performance in the WS-SSIM metric. Compared to the recently proposed method, BPOSR [24], although it has advantages in parameter count and computation, our method outperforms BPOSR by 0.057 on the WS-SSIM metric. Furthermore, the input size of BPOSR is 32 times larger than ours. In particular, in the 8× SR task, our method follows the design principle of OSTR with an input size of 32×32, while the input size of BPOSR is 128×256. It is worth noting that the input resolution is crucial in the SR task because it directly affects the model’s ability to perceive global information. Larger input sizes usually provide more details and contextual information, thus enhancing the model’s ability to capture global structures and details. Notably, even with this resolution disparity, our method outperforms BPOSR in both quantitative metrics and qualitative comparisons. The superior performance achieved with significantly smaller input patches highlights our model’s advantages concerning efficient information utilization and architectural design.

**Figure 9:**
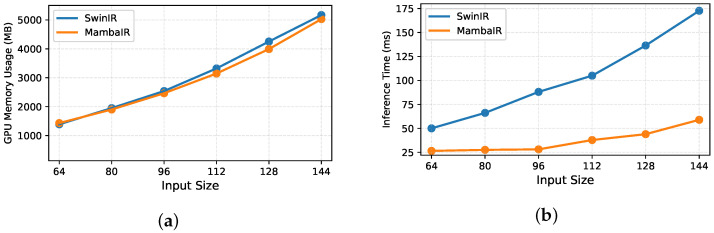
Comparison of (**a**) GPU memory consumption and (**b**) inference latency across varying input resolutions. We chose SwinIR (a Swin transformer-based method) and MambaIR (a Mamba-based method) for comparison. We adjust the model to ensure the GPU usage is roughly similar at the beginning, and then we increase the input resolution from 64×64 to 144×144.

To further demonstrate the superiority of the Mamba architecture over the transformer architecture, we conducted a comparative analysis between our baseline model, MambaIR, and the widely adopted ODISR baseline model, SwinIR, in terms of their computational efficiencies. As shown in Figure 9a, MambaIR exhibits linear GPU memory consumption with increasing input size, similar to SwinIR, which employs efficient attention mechanisms. However, it is noteworthy that MambaIR maintains a global receptive field comparable to standard full attention, while SwinIR’s receptive field is inherently limited by its window size. Regarding inference time, as illustrated in Figure 9b, experimental results demonstrate MambaIR’s significant efficiency advantages. Particularly, when the input image size increases to 144×144, SwinIR’s inference latency becomes approximately three times higher than that of MambaIR. These findings conclusively prove that the Mamba-based backbone network maintains global modeling capability and significantly outperforms the Swin transformer-based architecture in computational efficiency when processing large-scale input images.

### 4.4. Ablation Study

To gain a comprehensive analysis of MambaOSR, we conduct ablation studies on the effectiveness of each component in the proposed MambaOSR. To save time, all ablation experiments are based on modifications of the MambaIR-light model. The training dataset consists of 1200 omnidirectional images from ODISR [1], with an upscaling factor of ×8. The testing dataset includes 100 test images from ODISR [1] and 100 test images from SUN360 [46]. In the following experiments, we use the same framework and ensure that the parameters across different variants are approximately the same to eliminate the influence of parameter variations. We change only one component in each experiment.

#### 4.4.1. Effect of FAM

**Figure 10:**
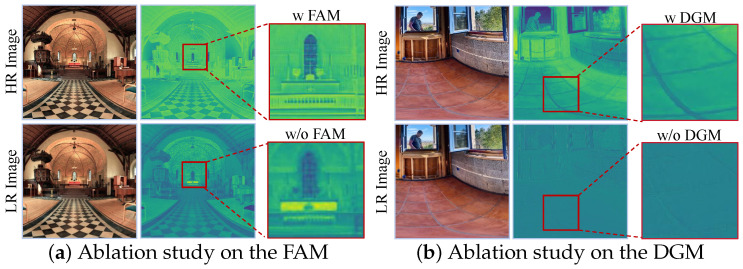
Comparison of feature maps in ablation studies. (**a**) Ablation study on the FAM: Feature maps with an FAM exhibit an enhanced ability to extract high-frequency information, resulting in sharper edges and finer texture details, while those without an FAM show blurred textures and loss of detail. (**b**) Ablation study on the DGM: Feature maps with a DGM effectively restore texture details in distorted regions, addressing projection distortions, whereas those without a DGM display limited recovery and less coherent structures.

To investigate the effectiveness of the FAM in ODISR, we conduct ablation experiments by removing it from MambaOSR. We replace the FFC layer with three consecutive 3×3 convolutional layers to maintain comparable parameter counts. As shown in Table 5, the FAM-ablated variant (Model-1) exhibits a 0.2 dB reduction in PSNR compared to the complete MambaOSR architecture. Furthermore, Figure 10a compares the intermediate feature maps of MambaOSR and its FAM-ablated variant, revealing that MambaOSR generates more detailed textures in complex regions, particularly in the window areas. In contrast, the variant without frequency-domain processing exhibits noticeable blurring in its intermediate features. These comparative results demonstrate that our FAM enhances high-frequency information extraction through frequency-domain analysis, thereby improving reconstruction quality.

#### 4.4.2. Effect of DGM

To evaluate the effectiveness of the DGM, we conduct an ablation study by replacing the DGM with a channel attention block in the MambaOSR architecture. In the ablated variant, we concatenate the distortion prior features with channel attention outputs along the channel dimension, followed by three 3×3 convolutional layers for feature fusion. As shown in Table 5, removing the DGM leads to a 0.36 dB decrease in PSNR compared to the MambaOSR architecture.

Furthermore, as illustrated in Figure 10b, in regions with severe geometric distortions (highlighted by red boxes), the DGM-ablated variant (Model-2) produces visible artifacts, while our MambaOSR successfully preserves fine texture details. These results demonstrate that the distortion-guided modulation enables adaptive feature processing based on local distortion patterns, facilitating more effective reconstruction in regions with varying distortion levels. The quantitative and qualitative improvements validate the effectiveness of our proposed DGM in handling geometric distortions in ODIs.

#### 4.4.3. Effect of MFFM

Through ablation studies where we remove the MFFM from MambaOSR (Model-3), we observe a 0.14 dB reduction in PSNR compared to the MambaOSR architecture. This performance degradation suggests that the MFFM effectively aggregates hierarchical feature representations across different scales. The module’s cross-scale integration capability provides complementary contextual information for the reconstruction process, enabling more effective utilization of multi-resolution features. As indicated in Table 5, quantitative comparisons demonstrate that this architectural component enhances texture preservation in high-frequency regions.

## 5. Conclusions

This work presents the first application of the Mamba architecture to ODISR tasks. By leveraging the long-range modeling capabilities of the VSSM and integrating spatial-frequency dual-domain insights into ODIs, we propose the MambaOSR framework. To overcome the limitations of conventional approaches in global modeling, we design the SFMB, which incorporates two key components: an FAM for global contextual reconstruction through representation learning, and a DGM for reducing geometric distortion artifacts. Specifically, the FAM enables effective global context modeling, while the DGM adaptively modulates features based on local distortion patterns derived from distortion prior maps, significantly improving reconstruction quality in severely distorted regions. To further enhance feature representation, we introduce an MFFM that facilitates cross-scale semantic integration during shallow feature extraction and high-level reconstruction stages. The extensive experimental results on multiple benchmark datasets demonstrate that our approach achieves state-of-the-art performance while maintaining competitive computational efficiency. It strikes an optimal balance between reconstruction quality and processing speed.

## Figures and Tables

**Table 1:** Definitions of symbols (and their meanings) used in Section 3.

Symbol	Description
SFMG	Spatial-frequency Mamba group
SFMB	Spatial-frequency Mamba block
SF-VSSM	Spatial-frequency visual state space model
FAM	Frequency-aware module
DGM	Distortion-guided module
MFFM	Multi-scale feature fusion module
φ,θ	Longitude and latitude
$Q_{s}=\left(q_{s_{x}},q_{s_{y}},q_{s_{z}}\right)$	Cartesian coordinates
h(·),t(·)	Coordinate transformation (sphere to 2D)
D(h,1:W)	Pixel stretching ratio at height *h*
$H,W,C_{in}$	Height, width, and channel dimensions
$\alpha_{l},\alpha_{l+1}$	Learnable scaling factors
$x_{in},x_{out}$	Input and output features of the MFFM
$z_{c}$	Channel-wise statistics
$W_{1},W_{2}$	Learnable weight matrices
h,w	Feature height and width
$w_{out}$	Branch weights in the MFFM
$f_{b},O$	Branch operation and total branches
σ(·)	ReLU activation function

**Table 2:** Quantitative comparisons (WS-PSNR/WS-SSIM) with SISR and ODISR algorithms on ODI-SR and SUN360 datasets, where the best results are highlighted in **red**. Note that our method follows OSRT by fixing the GT patch size to [256,256] during training, while BPOSR uses [1024,2048], with an LR patch size defined as [GTpatchsize/scale]. Most of the experimental data results in the table are derived from BPOSR [24]. Methods with a gray background indicate those based on the VSSM or a frequency-domain representation.

Dataset		ODI-SR						SUN360					
Scale		×4		×8		×16		×4		×8		×16	
Method		WS-	WS-	WS-	WS-	WS-	WS-	WS-	WS-	WS-	WS-	WS-	WS-
		PSNR	SSIM	PSNR	SSIM	PSNR	SSIM	PSNR	SSIM	PSNR	SSIM	PSNR	SSIM
SISR	Bicubic	24.62	0.6555	19.64	0.5908	17.12	0.4332	24.61	0.6459	19.72	0.5403	17.56	0.4638
	SRCNN	25.02	0.6904	20.08	0.6112	18.08	0.4501	26.30	0.7012	19.46	0.5701	17.95	0.4684
	VDSR	25.92	0.7009	21.19	0.6334	19.22	0.5903	26.36	0.7057	21.60	0.6091	18.91	0.5935
	LapSRN	25.87	0.6945	20.72	0.6214	18.45	0.5161	26.31	0.7000	20.05	0.5998	18.46	0.5068
	MemNet	25.39	0.6967	21.73	0.6284	20.03	0.6015	25.69	0.6999	21.08	0.6015	19.88	0.5759
	MSRN	25.51	0.7003	23.34	0.6496	21.73	0.6115	25.91	0.7051	23.19	0.6477	21.18	0.5996
	EDSR	25.69	0.6954	23.97	0.6483	22.24	0.6090	26.18	0.7012	23.79	0.6472	21.83	0.5974
	D-DBPN	25.50	0.6932	24.15	0.6573	22.43	0.6059	25.92	0.6987	23.70	0.6421	21.98	0.5958
	RCAN	26.23	0.6995	24.26	0.6554	22.49	0.6176	26.61	0.7065	23.88	0.6542	21.86	0.5938
	DRN	26.24	0.6996	24.32	0.6571	22.52	0.6212	26.65	0.7079	24.25	0.6602	22.11	0.6092
	HAT	26.52	0.7494	24.42	0.6759	22.61	0.6284	26.93	0.7854	24.26	0.7063	22.02	0.6395
	MambaIR	26.91	0.7595	24.46	0.6737	22.59	0.6263	27.58	0.7997	24.32	0.6998	22.06	0.6404
ODISR	360-SS	25.98	0.6973	21.65	0.6417	19.65	0.5431	26.38	0.7015	21.48	0.6352	19.62	0.5308
	LAU-Net	26.34	0.7052	24.36	0.6602	22.52	0.6284	26.48	0.7062	24.24	0.6708	22.05	0.6058
	SphereSR	–	–	24.37	0.6777	22.51	**0.6370**	–	–	24.17	0.6820	21.95	0.6342
	OSRT	26.89	0.7581	24.53	0.6780	22.69	0.6261	27.47	0.7985	24.38	0.7072	22.13	0.6388
	BPOSR	26.95	0.7598	24.62	0.6770	22.72	0.6285	27.59	0.7997	24.47	0.7062	22.16	0.6433
	FATO	26.78	0.7589	24.54	0.6784	**22.73**	0.6314	27.59	0.8035	24.42	**0.7120**	**22.18**	0.6449
	MambaOSR	**27.01**	**0.7616**	**24.62**	**0.6792**	22.66	0.6293	**27.75**	**0.8042**	**24.49**	0.7119	22.12	**0.6452**

**Table 3:** Quantitative comparison with state-of-the-art methods on ODI-SR and SUN360 datasets. PSNR/SSIM ↑: the higher, the better; LPIPS ↓: the lower, the better. LPIPS scores can better reflect texture quality; the best and second-best performances are marked in **red** and blue, respectively.

Method	Scale	ODISR			SUN360		
		PSNR↑	SSIM↑	LPIPS↓	PSNR↑	SSIM↑	LPIPS↓
360-SS [19]	×4	25.545	0.7251	0.3871	25.483	0.7123	0.4113
BPOSR [24]	×4	27.774	0.7812	0.3064	28.289	0.7966	0.2754
Ours	×4	**27.875**	**0.7839**	**0.2969**	**28.512**	**0.8021**	**0.2561**
LAU-Net [1]	×8	25.136	0.6953	0.4990	24.957	0.6967	0.4949
360-SS [19]	×8	22.762	0.6564	0.5541	22.452	0.6366	0.6061
BPOSR [24]	×8	**25.453**	0.7078	0.4631	25.339	0.7133	0.4596
Ours	×8	25.450	**0.7097**	**0.4442**	**25.359**	**0.7183**	**0.4245**

**Table 4:** Performance comparison of different methods on mutual information (MI↑, higher is better). The best results are highlighted in **red**.

Method	MI↑			
	MambaOSR	BPOSR [24]	LAU-Net [1]	360-SS [19]
ODI-SR	**2.6419**	2.6019	2.4884	2.0607
SUN360	**2.5568**	2.5068	2.3952	1.8534

**Table 5:** Ablation studies on MambaOSR components. All models are trained on the ×8 SR task under ERP downsampling and evaluated on ODI-SR and SUN360 datasets. The best results are marked in **bold**.

Model	FAM	DGM	MFFM	ODI-SR		SUN360		Params.
				PSNR	SSIM	PSNR	SSIM	(M)
Baseline	×	×	×	23.95	0.6650	23.82	0.6792	2.57
Model-1	×	✓	✓	24.33	0.6639	24.15	0.6859	2.59
Model-2	✓	×	✓	24.17	0.6508	23.99	0.6766	2.62
Model-3	✓	✓	×	24.39	0.6637	24.19	0.6860	2.60
MambaOSR	✓	✓	✓	**24.53**	**0.6747**	**24.38**	**0.7023**	2.60

## Data Availability

The original data presented in this study are openly available at the LAU-Net repository (https://github.com/wangh-allen/LAU-Net, accessed on 1 March 2025).
